# Aroma diversification and formation of bioactive tyrosol and tryptophol acetates by the yeast *Hanseniaspora vineae* during cider fermentation

**DOI:** 10.3389/fnut.2026.1828580

**Published:** 2026-05-26

**Authors:** Leandro Acevedo, Valentina Martin, Florencia Tourne, Adelaide Gallo, Laura Fariña, Eduardo Boido, Remi Schneider, Eduardo Dellacassa, Francisco Carrau

**Affiliations:** 1 Oenology and Fermentation Biotechnology Area, Food Science and Technology Department, Facultad de Química, Universidad de la Republica, Montevideo, Uruguay; 2 Oenobrands SAS, Montpellier, France; 3 Aroma Biotechnology Laboratory, Organic Chemistry Department, Facultad de Quimica, Montevideo, Uruguay; 4 Centro de Investigaciones Biomedicas, Departamento de Bioquimica, Facultad de Medicina, Universidad de la Republica, Montevideo, Uruguay

**Keywords:** aroma bioactive compounds, cider fermentation, non-saccharomyces yeasts, sensory analysis, apple juice

## Abstract

**Introduction:**

The fermentative performance and sensory impact of ten native *Hanseniaspora vineae* strains isolated from Uruguayan vineyards were evaluated for cider production.

**Methods:**

Strain selection was conducted through microfermentation trials in apple juice, leading to the selection of two strains based on complete sugar consumption and the absence of sensory defects. These strains were subsequently evaluated at pilot scale and compared with a commercial *Saccharomyces cerevisiae* strain. Chemical characterization of the flavor metabolome of the resulting ciders was performed using GC–MS and HPLC, together with sensory analysis based on CATA methodology.

**Results:**

Ciders fermented with the selected *H. vineae* strains showed a significantly enhanced production of volatile aroma compounds, particularly acetate esters derived from aromatic higher alcohols, compared to the commercial control. Notably, tyrosol and tryptophol acetates—compounds recently reported to exhibit anti-inflammatory bioactivity in animal models—were exclusively detected in ciders fermented with *H. vineae*, highlighting a distinctive metabolic trait of this non-*Saccharomyces* species. Sensory analysis associated *H. vineae* fermentations with fruity and citrus descriptors, consistent with their differentiated volatile profiles.

**Conclusion:**

This study demonstrates that native *Hanseniaspora vineae* strains diversify cider aroma and promote the formation of bioactive acetate esters, supporting their use as functional non *Saccharomyces* starters in cider fermentation. These esters are not produced by *Saccharomyces cerevisiae* under comparable conditions and, to our knowledge, are reported here in ciders for the first time.

## 1 Introduction

Research and development work into unconventional yeasts has become a strategic focus for the differentiation of fermented foods, especially in a market that demands ever-higher standards of sensory quality and safety. According to Market.us,^1^,^2^ the fermented food market is projected to grow globally from USD 573.4 billion (2024) to double in 2034, with an annual growth rate of 6.8%. According to this database, the cider market is growing at a comparable rate to the global fermented food market. In this scenario, interest in incorporating non-*Saccharomyces* yeasts to the production of fermented products has increased significantly, driven by their potential to generate unique aromatic profiles and provide attributes that increase sensory complexity and the presence of healthy bioactive compounds (1–3). Non-*Saccharomyces* yeasts have also emerged as promising alternatives in other fermented products, such as wine, due to their ability to produce a wide range of desirable enzymes and secondary metabolites that contribute to greater aromatic complexity and flavor diversity (4, 5). Besides, the continuous use of *S. cerevisiae* in cider production often results in limited differentiation in the sensory profile of ciders. Therefore, there is a growing need to identify and select yeast strains specifically adapted to cider fermentation, capable of contributing distinctive regional and sensory characteristics (6–9). Although with more limited reports than wines, in recent years several studies have searched for non-*Saccharomyces* yeast species in cider, with the aim of giving apple fermented products greater differentiation. Twenty native yeast species belonging to 10 genera were identified in apples or apple juice, including *Candida railenensis*, *Candida cylindracea*, *Hanseniaspora uvarum, Hanseniaspora meyeri*, *Hanseniaspora pseudoguilliermondii* and *Metschnikowia sinensis* (10). Some of the species tested at the laboratory level also include *Wickerhamomyces anomalus* (*Pichia anomala*), *Meyerozyma caribbica*, *Pichia kluyveri*, *Hanseniaspora uvarum* and *Starmerella bacillaris*, which increase the quality of ciders (11). A strain of *Pichia kluyveri* was the most suitable strain for Fuji apple juice fermentations. It generated prominent tropical aromas thanks to acetate esters as the main contributors (7). Some of these native yeast species showed low fermentative capacity on their own. However, with nutrients such as magnesium, zinc, and nitrogen, their ethanol production improved significantly, exceeding 80 g/L (6). Interestingly, some strains of *Williopsis saturnus* used in cider consumed few sugars, producing low alcohol contents (1.8–3% v/v), highlighting their potential for mild, low ethanol ciders (12), although semi-sweet. The production of cider from apple juice has been shown to be a significant source of *Hanseniaspora* species (13–16). In a previous work, *H. vineae* strains were selected for their capacity to produce cider through single-yeast fermentation processes, with the ability to yield up to 10% ethanol by volume (17). This represents an intriguing application of the species, as complete fermentation can be achieved using either single or mixed cultures of other non-*Saccharomyces* yeasts, without requiring the involvement of *Saccharomyces* strains. There are few studies investigating the use of *Hanseniaspora* species in cider fermentation; however, as observed in wines, always mixed fermentations with *Saccharomyces* have been associated to complete the fermentation (8, 18). Currently, the active dry wine strain *H. vineae* from our collection, marketed under the name Fermivin® VINEAE (Oenobrands), is being promoted as providing intense floral aromas, better texture and greater sensory complexity in various white wines. It has been tested in cider production, either in single inoculation or in coinoculation with *S. cerevisiae*, with preliminary very good results at the industrial level (data not shown). In wines, VINEAE strain stands out for its high production of phenethyl acetate (a rose-like floral aroma), fast lysis (which accelerates the improvement of the mouthfeel), does not produce undesirable compounds such as H_2_S and produces low levels of acetic acid (19). When applied to cider some preliminary results with *H. vineae* also demonstrated significant production of phenethyl acetate compared to *Saccharomyces* strains (18). A wide range of *H. vineae* strains has shown remarkable metabolic diversity. These strains can produce numerous aromatic compounds in wine musts, particularly benzenoids and acetate esters such as 2-phenylethyl acetate (20) and the acetates of tyrosol and tryptophol (3). This metabolic behavior contributes to their aromatic profile, differentiating them from those produced only with *S. cerevisiae* and other non-*Saccharomyces* strains under mixed cultures, which are usually classified as having a simpler aroma and more acidic taste (9). In addition, the production of other compounds, such as glycerol or manoproteins, can contribute to a better mouthfeel of the product (21, 22). Another potential advantage of *H*. *vineae* strains is their proteolytic activity (23), which could be of great help in clarifying the final product with minimal use of additives to prevent protein haze. This study aimed to evaluate the fermentative performance, enrichment of volatile bioactive compounds, and sensory impact of 10 *H. vineae* strains on cider production. A commercial *H. vineae* wine yeast (VINEAE) and a *S. cerevisiae* strain were used as controls.

## 2 Materials and methods

### 2.1 Yeast

*H. vineae* strains (Hv) were isolated from Uruguayan vineyards and belong to the collection of the Oenology and Fermentation Biotechnology Area of the Facultad de Química-UdelaR. Strains were previously selected for their ability to produce higher aroma diversity in a white wine model medium (1) and genetically differentiated by Martin et al. (20). The 10 strains of the *H. vineae* species (Table 1) were grown on plates with WL Nutrient Agar (Difco™, Becton Dickinson, Sparks, MD, United States) at 28 °C for 48 h. They were maintained on Petry dishes at 4 °C until use. As a control, two commercial yeasts were used: *H. vineae* Fermivin® VINEAE (Oenobrands, Montpellier, France), as Hv (VINEAE) and *S. cerevisiae* var. *bayanus* ILR 4F9 (Oenobrands, Montpellier, France, as Sc), both available as active dry yeasts and used following the manufacturer’s instructions.

**Table 1:** Native *Hanseniaspora vineae* yeast strains studied.

Assayed yeast codes	
02_19	12_151
02_25	12_184
11_24	12_196
11_48	12_219
12_111	12_213

### 2.2 Apple juice

In every fermentation trials, a commercial pasteurized Granny Smith apple juice (Pura Frutta™, Patagonia, Argentina) with no added nutrients was used. The initial physicochemical values before fermentation are: free amino nitrogen (FAN) of 75.09 mg N/L; pH of 3.41; total sugars of 123.12 g/L; and total acidity of 4.16 g/L, expressed as sulfuric acid.

### 2.3 Cider-micro fermentations at a laboratory scale

Micro fermentations at laboratory scale were carried out to evaluate the fermentative capacity of the different strains and their aromatic profile to select the strains for subsequent pilot-scale fermentations. Micro-fermentations were conducted in 250 mL flasks containing 150 mL of commercial apple juice, each inoculated with 1 × 10^6^ cells/mL of the yeast strains listed in Table 1. Inocula were prepared from single colonies from the WL Nutrient agar reactivated overnight in the same apple juice at 25 °C. Fermentations were conducted at 20 °C and monitored daily by measuring mass loss (mg/100 mL) due to CO₂ release. WL Nutrient agar was used also to check eventual contaminations during the processes. Each experiment was performed in triplicate, and the commercial yeasts detailed in Section 2.1 were used as controls.

### 2.4 Cider pilot scale fermentation

Two *H. vineae* strains were selected from lab-scale fermentations based on their lack of sensory defects, low volatile acidity, and higher fermentation rates compared to control strains. Pilot-scale ciders were produced with these strains in 3-liter fermenters using the same apple juice and control strains used in the micro fermentation stage, and with the same inoculum concentration of 1×10^6^ cells/mL. Fermentation was carried out in hermetically sealed vats equipped with an air trap at a temperature of 20 °C in triplicate. Fermentation was monitored by measuring the density with a portable EasyDens densimeter (Anton Paar, Graz, Austria). After fermentation, the ciders were transferred to full containers to allow yeast sedimentation. They were then left to chill (6 °C) for a week to achieve greater clarification before bottling. Finally, the ciders were placed in 500 mL bottles without filtration, after adding SO_2_ from a concentration of 60 mg/L of potassium metabisulfite and dimethyl dicarbonate (DMDC, Velcorin™, LANXESS Deutschland GmbH, Cologne, Germany) at a concentration of 0.1 mL/L. They were kept in a refrigerator at 6 °C until analysis.

### 2.5 Volatile and total acidity

The ciders produced were analyzed and evaluated with a 0.1 N NaOH solution until the phenolphthalein indicator changed color, according to the official AOAC 942.15 method (47). Total acidity was expressed in grams of sulfuric acid per liter. For volatile acidity, the cider was distilled using steam. The distillate was then titrated with 0.1 N NaOH according to the OIV method OIV-MA-AS313-02: R2015 (45). Volatile acidity is expressed in grams of sulfuric acid per liter.

### 2.6 Determination of organic compounds in ciders

Organic compounds were determined by HPLC, glucose, fructose, glycerol, and ethanol using the refractive index detector (RID) according to Perez et al. (24). Malic and acetic acids were determined using the UV absorption detector at 210 nm (25). Analytical standards were used for compound identification and quantification. Calibration curves in ultrapure water included concentrations for glucose and fructose (0.05–50 g/L), glycerol (0.02–6 g/L), ethanol (0.05–8% v/v), malic acid (0.1–14 g/L) and acetic acid (0.1–15 g/L). Results were expressed as g/L or % v/v as appropriate. All determinations were performed in duplicate, and a chromatogram processing was utilized. Separation was performed on a SUPELCOGEL C-610H column (30 cm × 7.8 mm, particle size 9 μm) maintained at 60 °C. The mobile phase used was 0.005 N sulfuric acid, with a flow rate of 0.5 mL/min.

### 2.7 Detection of volatile compounds by solid phase micro extraction (SPME)

The volatile profile of ciders obtained at pilot scale was analyzed by headspace solid-phase microextraction followed by gas chromatography–mass spectrometry (HS- SPME/GC–MS), following the methodology of Bingman et al. (26) with minor modifications. Briefly, 10 mL of cider were prepared for analysis in 20 mL vials with the addition of 2.4 g of NaCl and 10 μL of 2-octanol internal standard (concentration: 0.2786 g/L). In solid-phase microextraction, a fiber with the following phase characteristics was used for extraction: DVB/Carbon-WR/PDMS; thickness, 50/30 μm; and length, 10 mm (Restek, Bellefonte, PA, United States), then was carried out for 30 min at temperature 40 °C, under constant agitation (250 rpm). Volatile compounds were identified by gas chromatography coupled to mass spectrometry (GC–MS) using a Shimadzu QP-2010 Ultra system (Tokyo, Japan) equipped with a Stabilwax capillary column (30 m × 0.25 mm i.d., 0.25 μm film thickness; Restek Corporation, Bellefonte, PA, United States). Comparison of mass spectral fragmentation patterns with those stored on databases was also performed for compound identification (27).

### 2.8 Detection of tryptophol and tyrosol acetates by solid phase extraction (SPE)

A wider extraction of volatiles was performed by solid-phase extraction volatile organic compounds using an Isolute ENV + cartridge, (IST Ltd., Mid Glamorgan, United Kingdom), containing 1 g of highly cross-linked styrene-divinylbenzene polymer, as described by Dellacassa et al. (28). The free fraction was dried over anhydrous sodium sulfate and concentrated using a Vigreux column to a final volume of 4 mL. The extract was then reduced to 200 μL under a gentle nitrogen stream prior to GC–MS analysis. The chromatograms obtained were processed using LabSolutions software (Shimadzu Corporation, 2018, Japan), as described by Dellacassa et al. (28). Identification of volatile organic compounds (VOCs) was achieved by comparing the mass spectra of the samples with reference libraries (29–31) and by using linear retention indices and authentic standards.

### 2.9 Sensory evaluation of ciders

A sensory test of the pilot scale ciders was conducted with the participation of 32 semi trained panelists, 21 women and 11 men between 25 and 55 years old. The test was of the check-all-that-apply type (32) with an additional question about the quality of the cider analyzed using a 5-point hedonic scale ranging from low to high quality. The evaluation form was provided to participants via a QR code for virtual completion (Supplementary material) that includes an informed consent. The list of attributes used was initially obtained from previous literature (33) and then improved in an internal pilot test to add any terms that might be missing. The data were collected using the Compusense program (Compusense Inc., 2021). Subsequently, the statistical analysis was then performed using XLSTAT (Addinsoft, 2023).

### 2.10 Statistical analysis

ANOVA and post-hoc comparisons with Tukey’s test, using a significance value of 95%, were performed on concentrations of the aroma compounds resulting from fermentation and growth with *H. vineae*. ANOVA comparisons were performed using STATISTICA 7.0 software (StatSoft, Tulsa, OK). Differences in mean concentrations of aroma and non-volatile compounds were evaluated using the least significant differences test.

## 3 Results

### 3.1 Microfermentations in apple juice screening of *Hanseniaspora vineae* strains

**Figure 1:**
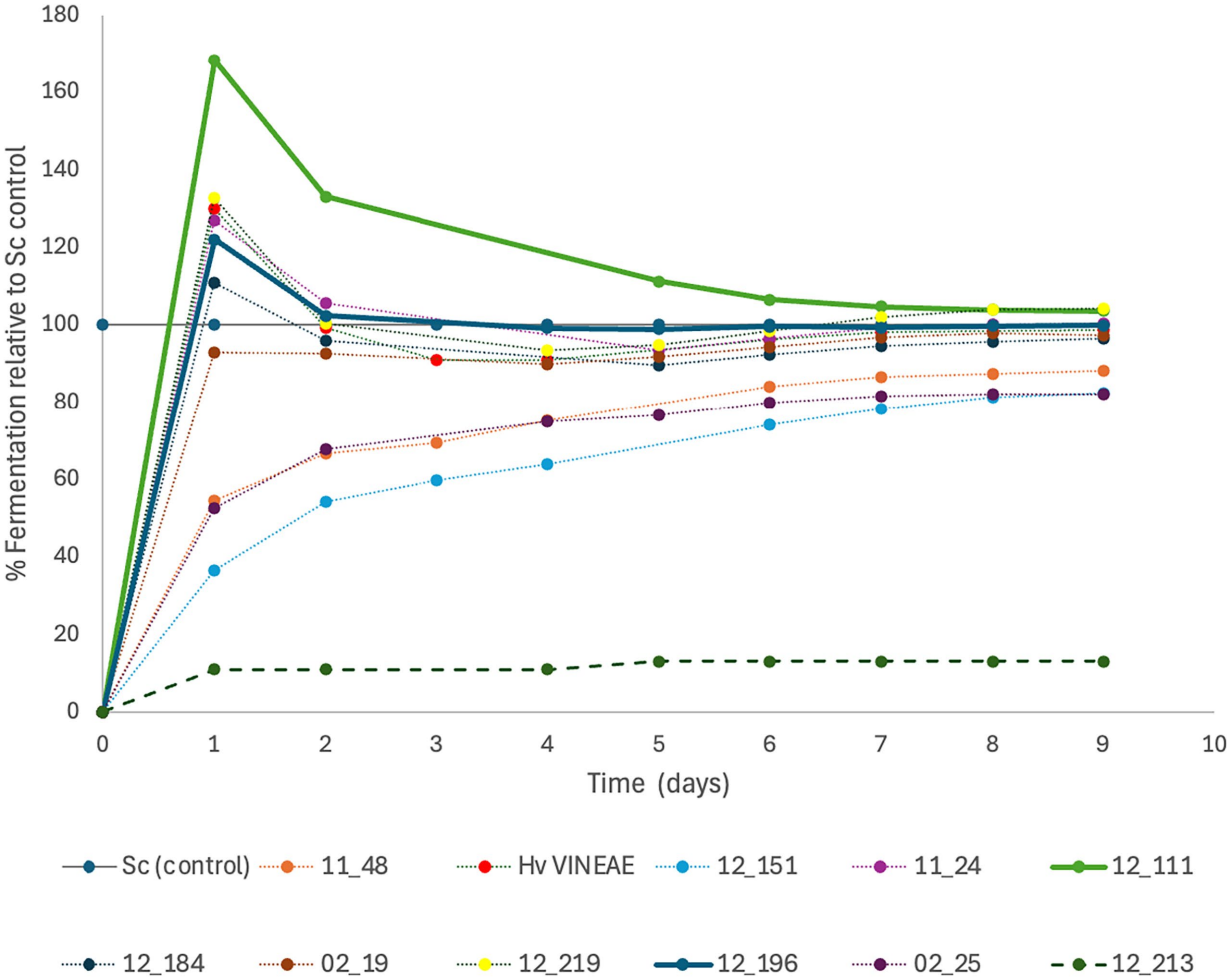
Mass loss measured by CO_2_ release from micro-fermentation flasks of the 10 *Hanseniaspora vineae* strains from the FQ Oenology collection and the *S. cerevisiae* control strain. Data were normalized to the *S. cerevisiae* control (100% relative fermentation behavior). *Hv* VINEAE is the commercial *H. vineae* control strain. Both selected *Hv* strains are shown as solid lines.

Micro fermentations were carried out with 10 Hv strains. Figure 1 shows the fermentation kinetics of the strains compared to the *S. cerevisiae* (ILR 4F9) and HvVINEAE control strains. Strain Hv12_213 was the only that not complete the fermentation process after 10 days. Strains Hv 12_196 and 12_111 were selected as the two best strains based on their fermentation performance, sensory quality (no defects) and preference with the internal tasting panel at the Oenology Laboratory (shown as solid lines in Figure 1). These two strains were then compared with the commercial strain HvVINEAE and *S. cerevisiae* at pilot scale level to enable a complete chemical and sensory characterisation of the final products.

### 3.2 Production of cider at pilot scale

**Figure 2:**
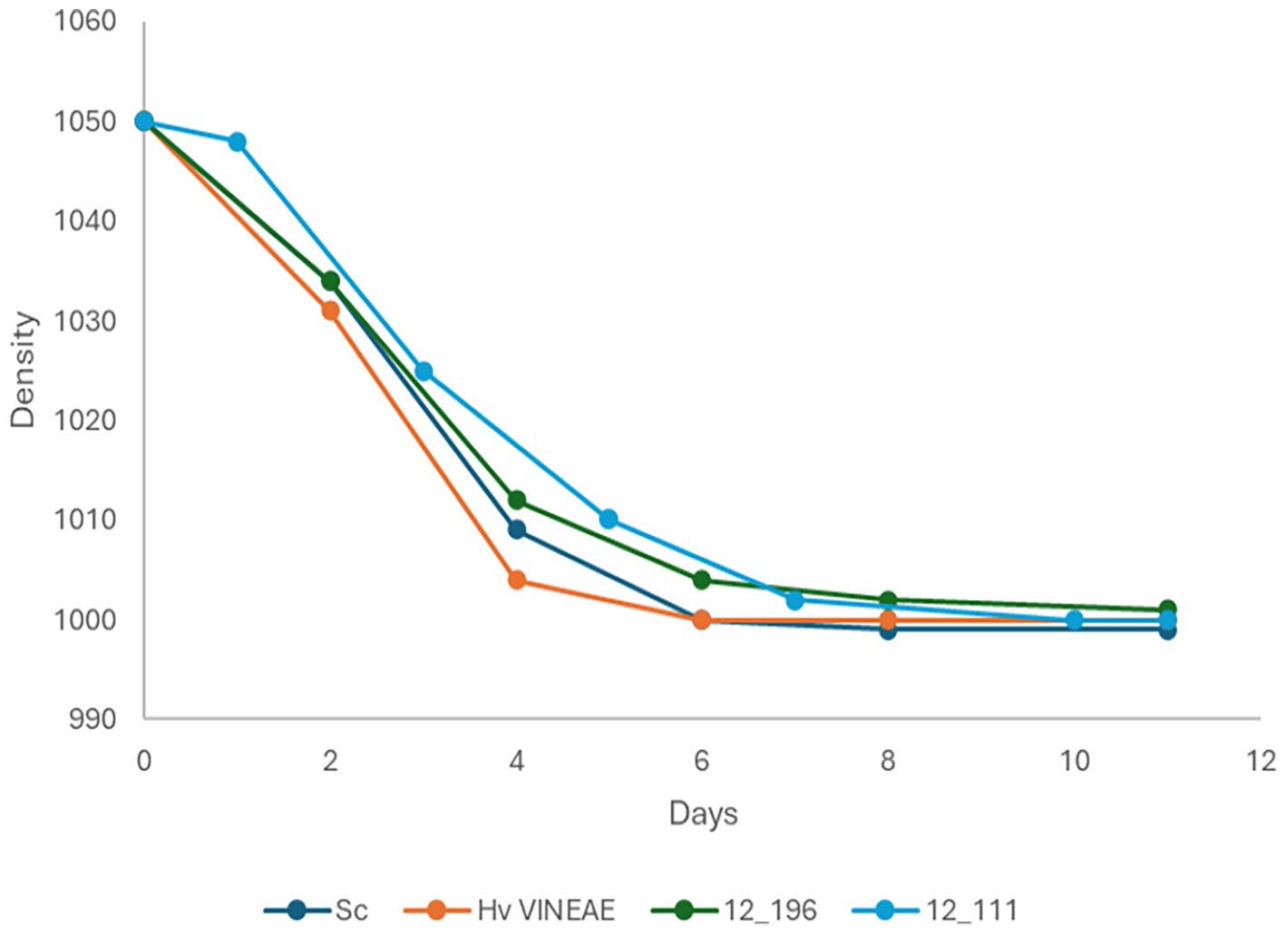
Monitoring of cider density over time in 3-liter pilot scale fermentations with the two selected strains of *Hv*.

Three-liter pilot fermentations of cider were produced using the selected strains and their respective controls. As shown in Figure 2, fermentations reached completion in approximately 10 days, with final densities near 1,000 for all batches. The two selected strains exhibited fermentation profiles similar to those of *Saccharomyces*, achieving final alcohol levels of 6.67–6.85% ABV (Table 2). Ciders fermented with the selected strains (Hv12_111 and Hv12_196) were notably clearer than those from the commercial *S. cerevisiae* and HvVINEAE controls; clarity was quantified by absorbance at 600 nm (Table 2).

**Table 2:** Physicochemical parameters of the juice and of the produced ciders with the two selected yeast *H. vineae* strains.

Parameter	Sc	12_196	12_111	Hv VINEAE	Apple juice
Final density	999	1,001	1,001	1,000	-
Absorbance at 600 nm	0.30b	0.19a	0.15a	0.47c	-
Total acidity (g H₂SO₄/L)	4.21 ± 0.08b	3.49 ± 0.03a	3.34 ± 0.04a	3.73 ± 0.03a	4.16 ± 0.01
Volatile acidity (g H₂SO₄/L)	0.40 ± 0.01a	0.49 ± 0.01ab	0.54 ± 0.01b	0.47 ± 0.01a	-
pH	3.48	3.63	3.76	3.60	3.41
Ethanol (% v/v)	6.95 ± 0.01b	6.67 ± 0.02b	6.85 ± 0.01b	6.80 ± 0.07b	NDa
Glycerol (g/L)	3.62 ± 0.29a	4.03 ± 0.02a	4.40 ± 0.08b	4.69 ± 0.07b	NDa
Glucose (g/L)	NDa	0.70 ± 0.02b	0.62 ± 0.01b	0.64 ± 0.01b	41.88 ± 0.73
Fructose (g/L)	NDa	1.13 ± 0.01b	2.23 ± 0.01b	NDa	81.24 ± 1.40
Malic acid (g/L)	6.01 ± 0.02b	5.18 ± 0.02a	4.69 ± 0.02a	4.95 ± 0.17a	7.00 ± 0.13
Acetic acid (g/L)	NDa	0.16 ± 0.01b	0.31 ± 0.01c	0.22 ± 0.01b	NDa

Sc, *Saccharomyces cerevisiae*, and Hv VINEAE control strains. Different letters showed significant differences between treatments in triplicate. ND, not detected.

### 3.3 Analysis of volatile acids, non-volatile acids, and sugars by HPLC of the ciders produced

Physicochemical parameters of the produced ciders are shown in Table 2. Total acidity was higher in the *S. cerevisiae* control cider than in ciders fermented with *H. vineae* (Hv) strains; Hv ciders showed a reduction in total acidity relative to the original juice. This lower acidity is attributable to significantly greater malic acid consumption by all Hv strains compared with the *S. cerevisiae* control, which also produced a slight increase in pH. Volatile acidity remained low and consistent across all samples, and both volatile and total acidity values fall within AOAC (34) quality cider limits. Residual sugars were higher in Hv fermentations than in *Saccharomyces* fermentations, indicating incomplete sugar consumption by Hv. HPLC analysis showed that Hv strains produced significantly more glycerol than the *S. cerevisiae* control.

### 3.4 Volatile compounds analysis by GC–MS

**Figure 3:**
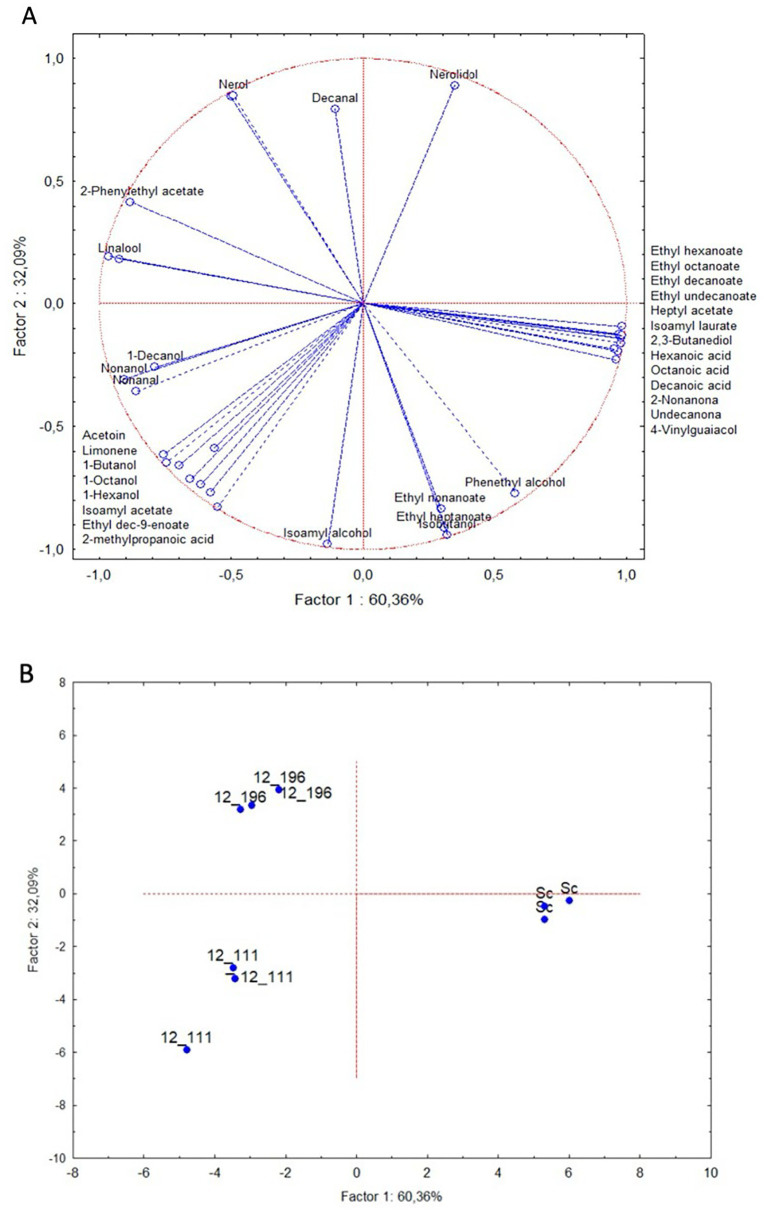
Principal component analysis (PCA) for the 42 aromas detected by GC in the pilot cider fermentations. **(A)** Shows the compounds and their discrimination, and **(B)** shows the strains in the analysis, clearly separated from *Sc. Saccharomyces*, 12_111 and 12_196 strains *H. vineae*.

Volatile compounds were analyzed; Table 3 lists the 42 compounds detected during fermentation with the selected strains. *H. vineae* (Hv) treatments showed a significant increase in acetates (isoamyl and 2-phenylethyl), ethyl decanoate, nonanol, and the monoterpene linalool. Conversely, both Hv fermentations had significantly lower concentrations of the main medium-chain fatty acids (hexanoic, octanoic, and decanoic acids) and their corresponding ethyl esters (including ethyl undecanoate and hexadecenoate). Two compounds characteristic of Hv fermentations but absent in *S. cerevisiae* controls—neryl acetate and acetoin—were detected; 4-vinylguaiacol was not detected in Hv fermentations. Principal component analysis of the 42 volatiles (Figure 3A) showed that the first two components explain 92.45% of total variability. The sample distribution in the component space (Figure 3B) separates Hv ciders (left quadrant) from the Sc control (right quadrant), indicating distinctly different aromatic profiles between Hv strains and Sc.

**Table 3:** Aroma compounds concentration by GC–MS of the produced ciders at pilot level with the two pre-selected *H. vineae* strains.

Compound (μg/L)	*Sc*	*12_196*	*12_111*
Esters			
Isoamyl acetate	56.83 ± 5.48 a	80.47 ± 6.64 b	150.16 ± 2.4 c
Ethyl hexanoate	253.65 ± 28.30 b	20.38 ± 3.12 a	43.39 ± 22.9 a
Ethyl heptanoate	3.54 ± 0.48 b	2.13 ± 0.11 a	3.66 ± 0.7 b
Ethyl octanoate	3546.13 ± 546.03 b	220.85 ± 53.55 a	448.98 ± 182.0 a
Ethyl nonanoate	5.19 ± 0.95 a	3.56 ± 0.18 a	5.41 ± 0.9 a
Ethyl decanoate	2227.81 ± 139.29 b	108.33 ± 46.47 a	153.20 ± 106.1 a
Ethyl dec-9-enoate	727.51 ± 125.37 a	821.17 ± 212.60 a	1641.28 ± 211.9 b
Ethyl undecanoate	191,15 ± 34,00 b	6,87 ± 2,12 a	17.52 ± 7.1 a
2-Phenylethyl acetate	13.62 ± 3.21 a	3997.41 ± 330.14 c	2393.15 ± 844.6 b
Heptyl acetate	1.72 ± 0.35 b	nd a	nd a
2-Phenylethyl propanoate	nd a	23.75 ± 1.39 b	nd a
Isoamyl laurate	8.28 ± 0.75 b	nd a	nd a
Ethyl hexadecenoate	11.37 ± 1.37 b	0.87 ± 0.12 a	nd a
Neryl acetate	nd a	6.43 ± 0.67 b	5.53 ± 1.6 b
Alcohols			
Isobutanol	31.28 ± 1.18 b	13.62 ± 0.40 a	35.21 ± 6.9 b
Isoamyl alcohol	1278.18 ± 11.77 ab	787.78 ± 71.89 a	1991.98 ± 579.4 b
1-Butanol	nd a	2.34 ± 0.38 b	9.91 ± 1.5 c
1-Hexanol	6.54 ± 0.34 a	7.56 ± 0.63 a	18.68 ± 7.7 b
1-Octanol	8.36 ± 0.53 a	9.45 ± 4.45 a	14.82 ± 1.8 a
1-Decanol	5.29 ± 0.09 a	10.95 ± 0.42 a	11.10 ± 1.3 a
Phenylethyl alcohol	155.67 ± 0.33 b	81.70 ± 7.72 a	137.78 ± 38.1 b
Nonanol	1.81 ± 0.05 a	11.75 ± 2.12 b	15.56 ± 1.2 b
2,3-Butanediol	3.61 ± 0.42 b	nd a	nd a
Carboxylic acids			
2-methylpropanoic acid	0.79 ± 0.18 a	2.23 ± 0.15 ab	6.67 ± 3.5 a
Hexanoic acid	19.37 ± 3.98 b	nd a	nd a
Octanoic acid	268.94 ± 36.95 b	16.00 ± 2.65 a	15.87 ± 2.8 a
Decanoic acid	126.64 ± 16.40 b	12.92 ± 4.52 a	5.78 ± 4.2 a
Dodecanoic acid	2.60 ± 0.07 b	nd a	nd a
Terpenes			
Linalool	1.87 ± 0.20 a	6.14 ± 0.48 b	5.49 ± 1.8 b
Limonene	nd a	nd a	11.47 ± 3.1 b
Nerolidol	3.44 ± 0.71 b	4.35 ± 0.18 b	nd a
Nerol	nd a	1.49 ± 0.15 b	nd a
Aldehydes			
Decanal	1.40 ± 1.10 ab	3.34 ± 1.54 b	nd a
Nonanal	nd a	1.53 ± 0.41 ab	2,67 ± 1.6 b
Ketones			
2-Nonanone	4.26 ± 0.70 b	nd a	nd a
Undecanone	4.46 ± 0.99 b	nd a	nd a
Acetoin	nd a	16.96 ± 5.83 b	133.94 ± 0.5 c
Phenols			
4-Vinylguaiacol	10.03 ± 1.22 b	nd a	nd a

Sc was the control cider strain of *S. cerevisiae*. Different letters showed significant differences between treatments in triplicate. nd, not detected.

### 3.5 Alternative extraction method for specific acetates by SPE

**Figure 4:**
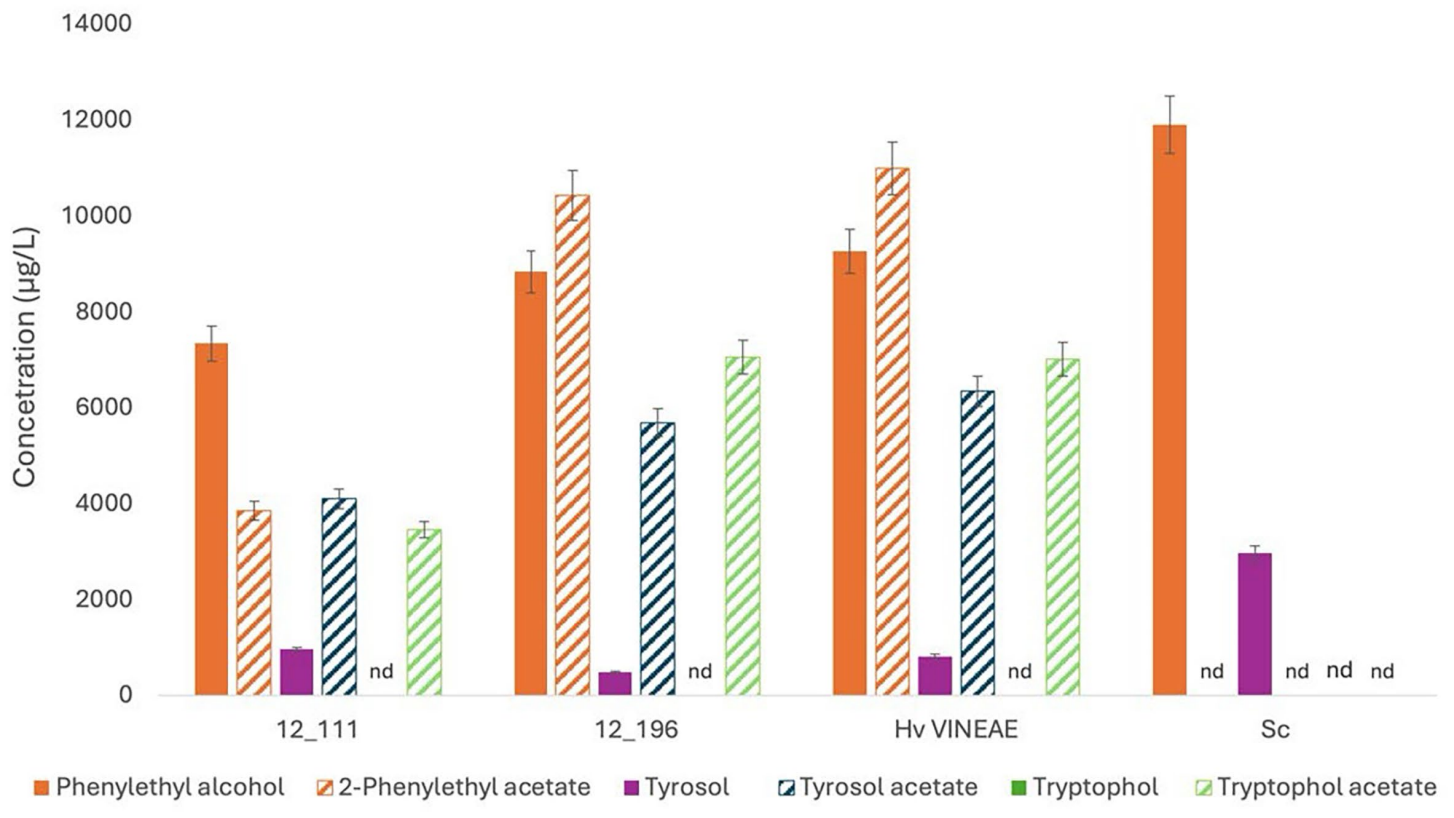
Concentrations of higher aromatic alcohols and their acetates in the tested samples; error bars represent standard deviation, and ND indicates not detected. *Sc*: *S. cerevisiae* indicating the absence of acetylation capacity of the three alcohols and the synthesis of tryptophol.

Compounds of interest, including tryptophol and tyrosol acetates (and their parent alcohols), were not detected by HS-SPME. Considering that the challenge of food metabolomics in relation to some volatile compounds is to search for different extraction methods (35), it was decided to use the SPE wine method for cider. Therefore, solid phase extraction (SPE) with ISOLUTE® ENV + cartridges was applied following Boido et al. (36), enabling detection of these compounds in ciders fermented with *H. vineae* (Hv) strains. As shown in Figure 4, strains 12_196 and Hv VINEAE produced comparable, higher levels of these acetates than strain 12_111. These acetates were absent from ciders fermented with the *S. cerevisiae* control, where only phenylethyl alcohol and tyrosol were detected. Notably, all tryptophol produced by the three Hv strains was found in its acetylated form, suggesting a highly efficient acetyltransferase activity in *H. vineae*. To our knowledge, this is the first report of tyrosol and tryptophol acetates in cider.

### 3.6 Sensory analysis of pilot-scale ciders

**Figure 5:**
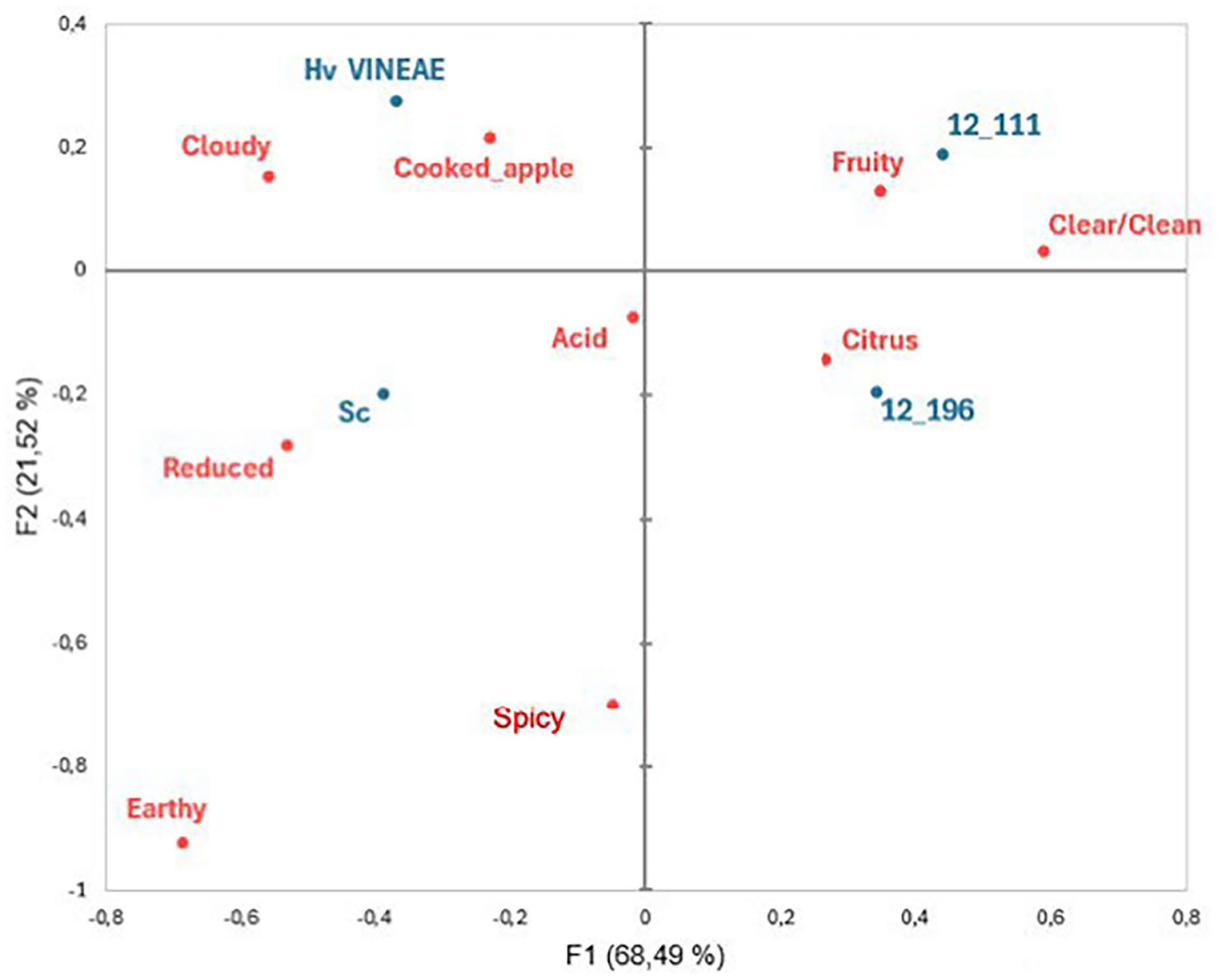
Graphical representation of the relationships between sensory attributes and yeast treatments in triplicate, using multidimensional correspondence analysis (MCA). *Sc*, *S. cerevisiae*, *Hv* strains 12_111 and 12_196, and *Hv vineae* commercial control.

A cider-specific tasting sheet was used and refined after preliminary trials by adding descriptors such as Vinegary/Acetic, Rose, and Honey (see Supplementary Figure). Correspondence analysis (Figure 5) shows strains 12_111 and 12_196 are characterized by clarity, citrus, and fruity notes, clearly separated from the controls (Sc and Hv VINEAE). Cider from 12_196 was more associated with citrus notes, while 12_111 was more associated with fruity notes. Table 3 confirms that Acidity was a prominent attribute in all ciders, with a significant predominance in the Sc control. Overall, the two Hv strains (12_111 and 12_196) contributed more fresh fruit and citrus aromas, and produced clearer, less acidic ciders than Hv VINEAE and the Sc control. In perceived quality (Table 4), 12_196 scored highest (mean 3.188/5), significantly outperforming the Sc control and the other Hv strains.

**Table 4:** Multiple pairwise comparisons using the critical difference procedure (Sheskin).

Attributes	*12_111*	*12_196*	Hv VINEAE	*Sc*
Cloudy	0.156 (a)	0.219 (a)	0.750 (b)	0.656 (b)
Clear	0.719 (b)	0.625 (b)	0.156 (a)	0.219 (a)
Floral	0.344 (a)	0.250 (a)	0.188 (a)	0.094 (a)
Fresh apple	0.281 (a)	0.406 (a)	0.188 (a)	0.156 (a)
Cooked apple	0.406 (ab)	0.250 (a)	0.594 (b)	0.531 (ab)
Cítric	0.219 (ab)	0.500 (b)	0.219 (ab)	0.188 (a)
Banana	0.125 (a)	0.156 (a)	0.094 (a)	0.250 (a)
Yeasty	0.125 (a)	0.188 (a)	0.281 (a)	0.219 (a)
Dry fruits	0.188 (a)	0.125 (a)	0.094 (a)	0.156 (a)
Vinegar/Acetic acid	0.156 (a)	0.188 (a)	0.344 (a)	0.188 (a)
Chemical/solvent	0.031 (a)	0.031 (a)	0.156 (a)	0.125 (a)
Earthy	0 (a)	0.031 (ab)	0 (a)	0.156 (b)
Frutal	0.500 (b)	0.406 (ab)	0.250 (ab)	0.219 (a)
Moldy	0.031 (a)	0.062 (a)	0.094 (a)	0.156 (a)
Sweet.	0.188 (a)	0.156 (a)	0.156 (a)	0.125 (a)
Bitter	0.125 (a)	0.094 (a)	0.125 (a)	0.062 (a)
Salty	0.094 (a)	0 (a)	0.062 (a)	0.125 (a)
Astringent	0.094 (a)	0.188 (a)	0.125 (a)	0.156 (a)
Reduced	0.031 (a)	0.062 (ab)	0.094 (ab)	0.219 (b)
Ácid	0.625 (ab)	0.719 (ab)	0.562 (a)	0.875 (b)
Alcoholic	0.094 (a)	0.156 (a)	0.219 (a)	0.156 (a)
Flavor with cherries	0.094 (a)	0.062 (a)	0.031 (a)	0.094 (a)
Caramel	0.156 (a)	0.094 (a)	0.094 (a)	0.062 (a)
Vegetal/Herbacous	0.156 (a)	0.250 (a)	0.125 (a)	0.188 (a)
Wine flavor	0.219 (a)	0.188 (a)	0.094 (a)	0.188 (a)
Honey	0.219 (a)	0.125 (a)	0.156 (a)	0.062 (a)
Spices	0 (a)	0.156 (a)	0.031 (a)	0.125 (a)
Rose	0.031 (a)	0.125 (a)	0.062 (a)	0.031 (a)

Different letters showed significant differences between treatments in triplicate.

## 4 Discussion

The results demonstrate that native *H. vineae* strains can diversify cider aroma and promote formation of bioactive acetate esters, enabling development of functional yeast starters for producing moderate-alcohol ciders with complete sugar consumption. The cleaner appearance observed with the two selected Hv strains warrants further study to identify the mechanism. This effect could reflect higher flocculation capacity, as reported in white wines (17), or enhanced protease activity previously described for *H. vineae* and linked to reduced bentonite demand (23). Further investigations will be carried out to assess this aspect (Table 5).

**Table 5:** Average quality perception for each variety.

Strain	Media (Cider Quality)	Group	
12_196	3,188	A	
12_111	3,031	A	B
Hv VINEAE	2,625	A	B
Sc	2,406		B

Grouping by Tukey’s test to establish significant differences. According to this analysis strain 12_196 was selected as for its significant differences with the conventional cider strain Sc.

Greater malic acid consumption by Hv strains—also reported in wine studies (37, 38)—accounts for the reduced total acidity and slight pH increase. The absence of lactic acid bacteria in the medium excludes bacterial malic conversion as a cause. No lactic acid was detected in ciders by HPLC analysis.

Many volatile compounds differed significantly between Hv strains and commercial starters. Elevated acetates, acetoin, and reduced medium-chain fatty acids (and their ethyl esters) are consistent with *H. vineae* behavior in grape must fermentations (37). High 2-phenylethyl acetate production—a hallmark of *H. vineae*—was observed and likely contributes floral/rose notes to cider (18, 37). While phenylethanol often accumulates in *Saccharomyces* fermentations, many *S. cerevisiae* strains do not convert it efficiently to the corresponding acetate ester that strongly influences aroma (20).

Using SPE (ISOLUTE® ENV+), tyrosol and tryptophol acetates were detected for the first time in ciders fermented by Hv, whereas the *S. cerevisiae* control produced only the precursor alcohol tyrosol (Figure 4). In the three Hv strains, tryptophol was found fully acetylated, suggesting an efficient specific acetyltransferase activity in *H. vineae*. Genomic differences—*S. cerevisiae* having fewer acetyltransferase gene copies (2) than *H. vineae* (5 gene copies)—may explain this capacity (39). These acetates likely have lower sensory thresholds than their alcohol precursors, so Hv-mediated acetylation is expected to enhance aroma impact. Although formal odor thresholds remain undetermined, preliminary sensory descriptions at concentrations found in wines report fruity and floral notes, with a higher aroma impact by the tyrosol acetate than the tryptophol acetate (40).

Tyrosol and tryptophol acetates are also of interest for bioactivity. Malka et al. (41) reported anti-inflammatory and anti–*Vibrio cholerae* effects at doses (150 μg/kg in mice) that suggest the concentrations observed here [comparable to values reported in Hv wines: ~5,000–10,000 μg/L; (40)] could be nutritionally relevant under moderate consumption. The precursor alcohols (tyrosol, tryptophol) additionally possess antioxidant and cardioprotective properties (46), and recent work shows anti-inflammatory activity *in vitro* (42). Note that the three aromatic amino acids are essential nutrients for animals that may derive from yeast metabolism or apple juice baseline levels (3).

Because these aromatic higher alcohols and their acetates appear to be produced uniquely by *H. vineae* (and by *K. marxianus* in kefir as it was mentioned) (43, 44), their detection in cider highlights both analytical challenges and the distinct metabolic potential of Hv starters. Future studies should (i) clarify mechanisms behind improved clarification, (ii) determine sensory thresholds for tyrosol and tryptophol acetates, and (iii) evaluate potential nutritional/bioactive effects of these compounds in cider and wine at concentrations achieved by *H. vineae* under realistic consumption scenarios.

## 5 Conclusion

The selected strains, 12_196 and 12_111, demonstrate fermentation capabilities comparable to Sc in ciders. In pilot-scale cider production, both strains performed similarly, completing fermentation within the same timeframe of 8 days. Chemical aroma characterisation using GC–MS revealed a differentiated aromatic profile compared to Sc, with notable compounds such as 2-phenylethyl acetate, isoamyl acetate, tryptophol and tyrosol acetate contributing to fruity aromas. Furthermore, many of these compounds have been shown not to be produced by *Saccharomyces* strains and are important bioactive compounds with anti-inflammatory properties that are beneficial to human health. These compounds have been reported in ciders for the first time here. The Hv strains produce these compounds uniquely or in greater quantities, with strain 12_196 standing out above the other two. Sensory analysis showed that ciders produced using Hv strains have a different sensory profile to those produced using the control commercial strains Sc and HvVINEAE. According to the results of the CATA sensory test, ciders produced with strain 12_196 have a more citrusy profile, while those produced with strain 12_111 have a fruitier profile. Sensory testing indicates that strain 12_196 is perceived as significantly higher in quality than the control strain Sc, although no significant differences were observed with respect to the other Hv strains. Another notable advantage of strains 12_196 and 12_111 is their natural ability to clarify (increase flocculation or protease capacity), which could reduce the need for additional clarification treatments during cider production. In terms of acidity, ciders produced with strains 12_196 and 12_111 were perceived as less acidic than the Sc control strain in the sensory test. This perception coincides with the results of the total acidity and malic acid content analyses. In addition, all Hv strains produced more glycerol than the control, which could make ciders produced with these strains taste fuller. In conclusion, strain 12_196 is the best candidate for producing ciders with different sensory profiles to those produced with *S. cerevisiae* or the commercial wine strain of the same species (Hv VINEAE). Future studies in relation to the assimilable and functional activity in human nutrition of the higher aromatic alcohols and their esters produced by *H. vineae* during fermented beverages should be studied to understand their food healthy potential of these compounds.

## Data Availability

The datasets presented in this study can be found in online repositories. The names of the repository/repositories and accession number(s) can be found in the article/Supplementary material.

## Footnotes

1
https://market.us/

2
https://market.us/report/fermented-foods-market/#utm_source=chatgpt.com
