# Supplementary material for: Aroma diversification and formation of bioactive tyrosol and tryptophol acetates by the yeast Hanseniaspora vineae during cider fermentation
Source: Front Nutr. 2026 May 26;13:1828580. doi: 10.3389/fnut.2026.1828580 (PMC13246365; doi:10.3389/fnut.2026.1828580)
Supplement: Supplementary file 1 [file Data_Sheet_1.pdf]

Supplemental data

Tasting sheet for ciders Acevedo et al.

You will be presented with four samples of cider to taste individually (one at a time). Smell the aroma and then take a sip to taste the flavor. Then discard the sample in the designated container. After tasting, complete the ballot. Cleanse your palate with water between samples.

Sample: Date:

Indicate the quality of the cider you tasted on the following scale

lower quality ☐ ☐ ☐ ☐ ☐ higher quality

Check all the words you think are appropriate to describe this sample of cider:

☐ Cloudy	☐ Chemical/solvent	☐ Alcohol
☐ Clear/clean	☐ Earthy	☐ Beer flavor
☐ Floral	☐ Fruity	☐ Caramel
☐ Fresh apple	☐ Musty	☐ Vegetable/herbaceous
☐ Cooked apple	☐ Sweet	☐ Wine flavor
☐ Citrus	☐ Bitter	☐ Honey
☐ Banana	☐ Salty	☐ Spicy
☐ Yeast	☐ Astringent	☐ Rose
☐ Dried fruit	☐ Reduced	
☐ Vinegary/acetic	☐ Acidic	
